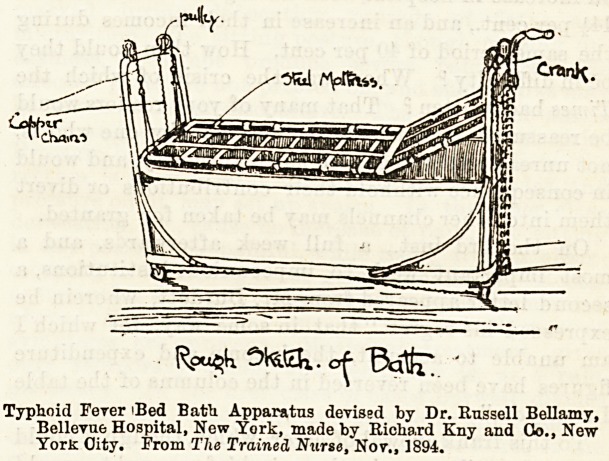# A Typhoid Fever Bath

**Published:** 1895-01-12

**Authors:** 


### PRACTICAL DEPARTMENTS.

### A TYPHOID FEVER BATH.

In our American contemporary, *The Trained Nurse* for November, 1894, Dr. Russell Bellamy, of the Bellevue Hospital, New York, gives a description of an apparatus for administering baths to typhoid patients, devised by himself and made by Richard Kny and Co., of New York City, which he finds accomplishes this treatment with the least possible shock to the patient and a minimum of attendance. We reproduce below a drawing of the bath in question from a sketch given in his article, which clearly shows the system upon which it is worked. The bath is of white enamelled iron—though any other material may be substituted if desired—6 feet 4 inches long, 22 in. deep, and 20 inches wide. An iron frame on rubber wheels supports the tub itself, and it is supplied with a syphon exhaust pipe, allowing every drop of water to be removed in a few minutes. Four strong copper chains pass over pulleys at the four corners of the frame, and over similar ones under the bath, supporting a comfortable steel mattress. "These chains are connected with an endless screw by cogs and a bicycle stop chain. The mechanism is controlled by a crank, so that the mattress can be raised or lowered by reversing the wheel. The apparatus is so arranged that the mattress can be raised several inches above the top of the tub or lowered to the bottom." A piece of white enamelled iron, 2 feet long by 6 inches wide, is used to bridge over the space between the bath and the bed when brought up for use.

The steel mattress is detachable, and thorough disinfection is possible. The whole is coated with white enamel.

The method of using this apparatus we give in Dr. Bellamy's own words: "The patient, naked and covered by a linen sheet, is placed upon a heavy rubber blanket which is perforated. The bath tub is brought to the bedside, and the nurse standing on the outer side . . . and gently drawing the sheet, brings first the patient's head, then body, and lastly feet upon the steel mattress. A comfortable rubber pillow is placed under the head. The crank is reversed, the patient lowered into the water suddenly or by degrees. The bath given, the mattress raised to the level of the bed, and the water having escaped through the holes in the rubber blanket, the patient is transferred to the bed in the same manner in which he was removed to the bath."

Dr. Bellamy has thought out this plan after a large experience in cases of typhoid fever in the wards of the Bellevue Hospital, in which the method known as "Brand's" yielded the best results, but which, requiring sometimes as many as five nurses—two for night, two for day duty, and a fifth assisting at intervals—for the efficient administration of

Typhoid Fever Bed Bath Apparatus devised by Dr. Russell Bellamy, Bellevue Hospital, New York, made by Richard Kny and Co., New York City. From *The Trained Nurse*, Nov., 1894.

constant baths, was naturally impossible in many instances, especially in private practice. Three or four nurses would be needed to transfer the patient from bed to bath in the ordinary course, and the return to bed was usually accomplished with even greater difficulty. By the use of Dr. Bellamy's mechanism one attendant is perfectly able to carry out the treatment, with far less disturbance, "mental or physical," to the patient, and with much modification generally of fuss and trouble. The particular "bath tub" described and illustrated in *The Trained Nurse* was especially designed for hospital use, but height and size are, of course, details to be decided upon as may best suit individual cases.

Dr. Bellamy has thus succeeded in materially simplifying the use of this method of treatment, for which singularly successful results are claimed, and has so brought it within reach in many cases where under former conditions it would have been most difficult, if not hopeless, of accomplishment.

## Figures and Tables

**Figure f1:**